# WebGIS-Based Real-Time Surveillance and Response System for Vector-Borne Infectious Diseases

**DOI:** 10.3390/ijerph20043740

**Published:** 2023-02-20

**Authors:** Momna Javaid, Muhammad Shahzad Sarfraz, Muhammad Umar Aftab, Qamar uz Zaman, Hafiz Tayyab Rauf, Khalid A. Alnowibet

**Affiliations:** 1 Department of Computer Science, National University of Computer and Emerging Sciences, Islamabad, Chiniot-Faisalabad Campus, Chiniot 35400, Pakistan; momina.javaid03@gmail.com (M.J.); shahzad.sarfraz@nu.edu.pk (M.S.S.); ms.umaraftab@yahoo.com (M.U.A.); qamar.zaman@nu.edu.pk (Q.u.Z.); 2 Independent Researcher, Bradford BD8 0HS, UK; 3 Statistics and Operations Research Department, College of Science, King Saud University, Riyadh 11451, Saudi Arabia; knowibet@ksu.edu.sa

**Keywords:** Geographical Information System, machine learning, vector-borne disease, climate, WebGIS

## Abstract

The diseases transmitted through vectors such as mosquitoes are named vector-borne diseases (VBDs), such as malaria, dengue, and leishmaniasis. Malaria spreads by a vector named Anopheles mosquitos. Dengue is transmitted through the bite of the female vector Aedes aegypti or Aedes albopictus mosquito. The female Phlebotomine sandfly is the vector that transmits leishmaniasis. The best way to control VBDs is to identify breeding sites for their vectors. This can be efficiently accomplished by the Geographical Information System (GIS). The objective was to find the relation between climatic factors (temperature, humidity, and precipitation) to identify breeding sites for these vectors. Our data contained imbalance classes, so data oversampling of different sizes was created. The machine learning models used were Light Gradient Boosting Machine, Random Forest, Decision Tree, Support Vector Machine, and Multi-Layer Perceptron for model training. Their results were compared and analyzed to select the best model for disease prediction in Punjab, Pakistan. Random Forest was the selected model with 93.97% accuracy. Accuracy was measured using an F score, precision, or recall. Temperature, precipitation, and specific humidity significantly affect the spread of dengue, malaria, and leishmaniasis. A user-friendly web-based GIS platform was also developed for concerned citizens and policymakers.

## 1. Introduction

Vector-borne diseases (VBD) are infectious diseases caused by any blood-sucking vector such as a mosquito, sandfly, etc. These diseases include Malaria, dengue fever, leishmaniasis, Zika, chikungunya, etc. [1]. These infections are increasing the disease burden in the world. More than 80% of the world’s lives are in danger due to the spread of the VBDs taking 700,000 people’s lives each year [2]. Pakistan is a sub-tropical country, and VBDs mainly spread in this region. VBDs are seasonal diseases. Pakistan is under the burden of many such devastating diseases. Malaria, dengue, leishmaniasis, etc. are some of these [2].

Malaria is the second most catastrophic disease after Acute Respiratory tract Infections (ARIs) in Pakistan [2]. Malaria is transmitted by a vector named genus Anopheles mosquitoes. Usually, the symptoms of Malaria are fever, cough, sweating, pain, diarrhea, headache, vomiting, chills, weakness, restlessness, back pain, splenomegaly, etc. [3], and it even leads to death [4]. In an area, climate change, poor sanitation, migration, etc., are some of the significant causes of malaria [5]. The reported confirmed cases were 374,510 in 2018, 413,533 in 2019, and 371,828 in 2020 [6]. The reported deaths were 511, 544, and 454 in 2018, 2019, and 2020, respectively, by the World Health Organization (WHO) [7].

Another devastating VBD is dengue. It is transmitted by the bite of female vectors Aedes aegypti or Aedes albopictus mosquito. It can spread in areas where poor sanitation, many refugees, overpopulation, or standing water in containers exist [8]. There is no proper treatment for this disease. Early detection and appropriate medical treatment can reduce the risk of severe dengue. Precautionary measures can help reduce the risk of severe dengue and lower the death rate to below 1% [9]. The identification of dengue is by any of the two listed symptoms: headache, muscle/joint pain, retro-orbital pain, positive tourniquet test, rash, and any warning signs of severe dengue, which include abdominal pain, high hematocrit value, persistent vomiting, swelling, mucosal bleeding, and liver enlargement [10] and can lead to death [8]. In 2019, 56,000 cases and 95 deaths have been reported for dengue all over Pakistan, of which 43% of cases and 23% of deaths were reported from Islamabad and Rawalpindi [2].

Leishmaniasis disease does not have the same importance as Malaria and dengue in Pakistan. The rising cases of leishmaniasis in Pakistan need to be highlighted. The cause of this ignorance may be due to a lack of education, poor health facilities, low standards of living, and lack of awareness [2]. The female genus Phlebotominae sandfly is the vector that transmits this disease [11]. The types of leishmaniasis are zoonotic and anthroponotic cutaneous leishmaniasis (CL) and zoonotic and anthroponotic visceral leishmaniasis (VL). CL can cure itself after some time, but it leaves scars on the skin. At later stages, VL becomes incurable [12]. The number of cases of cutaneous leishmaniasis reported was 19,361 in 2018, 53,574 in 2019, and 16,770 in 2020 [13].

The best way to control these VBDs is to identify those locations that are favorable breeding sites for their vectors. This can be efficiently accomplished by the Geographical Information System (GIS) [14]. The data processed in GIS have a unique identifier for each example. That unique identifier represents the location on earth [15]. GIS is helping today draw buffers and spatial parameters and helps collect climatic and environmental data [16]. Many studies are using GIS to solve their problem. A few examples are explained here. Researchers are finding the locations in agricultural land for land improvements and to increase utilization [17]. Another example from disaster management is finding the obstructions in the way of real-time disaster management of cyclones and risk analysis and management [18]. The third example is tourism. GIS has contributed to the creation of detailed sectional maps and the development of geographical tourism information databases [19].

**Figure 1:**
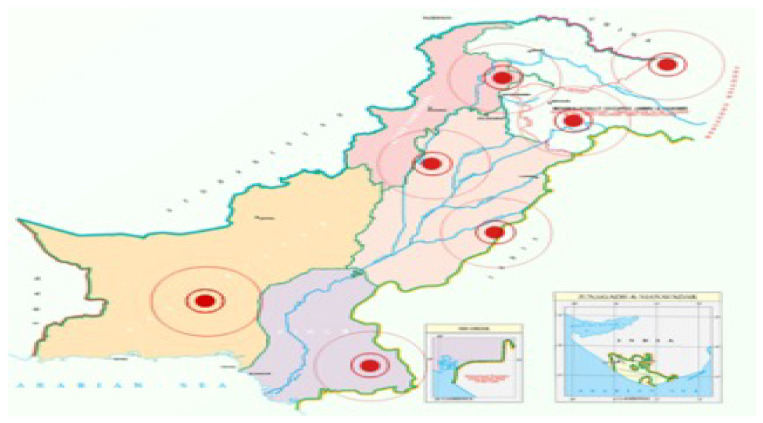
COVID-19 geographical risk map of Pakistan.

Today’s most common example of GIS is the COVID-19 website in Pakistan https://covid.gov.pk (accessed on 1 October 2021). Figure 1 shows the map of Pakistan, which is displayed on the website. The red circles are the warning signals for the high-risk areas of COVID-19. This website is freely available on the internet. Citizens of Pakistan can see the situation in their area at any time. The visualization on the map is easily understandable by non-technical people and policymakers. This website is helpful for the government in making decisions about preventive measures to save people from COVID-19.

The GIS helps visualize our geographical outbreak datasets easily and better understand plot epidemiology datasets on virtual globes using risk analysis and statistical techniques [16,20]. These techniques can contribute to identifying factors behind epidemics other than giving the geographical representation of risk [20]. The availability of geospatial disease datasets can not only be of great help to the concerned citizens but also to the policymakers. The near-real-time monitoring systems using GIS with Remote Sensing (RS) give accurate and updated Early Warning Systems (EWS) [16]. The techniques of GIS and RS collectively have proven to be effective methods for finding the vectors such as spatial analysis software to analyze vulnerable areas because geospatial technologies produce efficient and accurate results in epidemic monitoring. High-resolution satellite data with spatial information are currently very helpful in making urban environment strategies. Many applications have been developed using RS and GIS to create risk maps of malaria, dengue, or other epidemics [14,21]. GIS can be helpful in a manner that it can point out the exact location of the patient so that we can find the infected area and, by drawing it on the map, it can easily be understood [21].

Collectively predicting high-risk breeding habitats of malaria, dengue, and leishmaniasis using machine learning in real time is a challenging task. It is imperative to find the impact of each climate factor on each disease for disease control measures. With GIS mapping, it is easier for decision-makers to analyze the impact of each climatic factor on disease transmission in a geographical area. There is a need for the real-time prediction of diseases for immediate measures. These types of user-friendly web applications relating to VBDs in Pakistan do not exist on the internet. The research contributions of this study are:

- To conduct an extensive literature review of the relevant studies to identify which climate factors are the most important in vector-borne diseases’ spread.
- To find the relationship between climatic factors and diseases.
- To extract near real-time climate factors data to predict accurate results.
- To use malaria, dengue, leishmaniasis incidences, and meteorological factors to predict the outbreak in real time, using machine learning.
- To develop a user-friendly web-based GIS platform for real-time monitoring breeding habitats for the concerned citizens and policymakers.

The remaining sections of this paper consist of the following sections: Section 2 consists of a detailed literature review to identify the existing methods, tools, and techniques related to predicting VBDs. Section 3 is a detailed research methodology. This part discusses the step-by-step procedure, discussion on the dataset, the prediction models, and the development of WebGIS. Section 4 explains the results and discusses the predicted results of machine learning models and the best model selected for WebGIS. The results generated from WebGIS are also discussed. Section 5 contains the conclusion, and finally, Section 6 contains the future work of this research.

## 2. Related Work

This section includes an extensive literature review of the relevant studies to identify which climate factors are the most important in vector-borne diseases’ spread and also focuses on the machine learning models used in previous studies related to the problem discussed in Section 1.

The study [22] was conducted by including a survey of 92 villages in Phitsanulok Province of Thailand to analyze how land use/land cover is related to the dengue spread using spatial and statistical methods. The climatic conditions used in the study were temperature, humidity, and rainfall. This study used the spatial and temporal relationship between vector larval density and land useland cover to focus on identifying the potential breeding habitats of dengue mosquitoes while also identifying the land-use types and dengue indices. The estimates of vector population were completed by dengue indices such as house index, container index, and breteau index; these were used to find the relationship of each index with the habitat factors. The relationship between dengue cases and climatic conditions showed that climate factors play an essential role in the spread of dengue fever. Land cover can be an important factor in dengue transmission. Limitations included that the shape, size, and number of containers differed in all houses. It might have been the cause of the variation between the house index, container index, and breteau index.

The author of [23] used the climate (temperature, rainfall, and humidity) and air network traffic data to create a VBD (malaria, dengue, yellow fever, and chikungunya) website to observe the risk of transmission of VBDs through traveling using Climatic Euclidean Distances. The author pointed out that air traveling plays an important role in transferring VBDs through the infected travelers rather than the vectors. The contribution of this study was that the web portal was developed to monitor the VBDs’ attacks and the movement of the vectors. The author recommended some website features for future research: (1) regular updates about disease incidences; (2) updates about flights’ capacities and route data; (3) provide controlling and reduction guidance that will help people lessen the burden of outbreaks; (4) find the airport watershed areas and predict those areas that can be majorly affected by the imported disease instances or vectors through the help of different datasets; and (5) add more diseases (leishmaniasis, rift valley fever, and Chagas disease) to the portal.

The author [24] has performed fuzzy logic techniques to predict risk maps of Thailand’s dengue cases. Temperature, rainfall, humidity, land use/land cover, population density, elevation, and entomological data (larval density of dengue mosquitoes inside and around houses) were collected and used as factors affecting dengue transmission. The addition can make further improvements to new layers of infrastructure and socioeconomic factors. The results extracted at the local scale may not be used to generalize to the regional scale because factors influencing dengue vector densities vary due to ecological and social factors. The remote sensing data are according to the geospatial data of epidemiology.

The spatial analysis, ArcGIS, and patient and satellite data were used to study spatial distribution patterns of malaria in the district of Rawalpindi. The authors [25] made a comparison between different techniques for spatial and temporal analysis. A supervised classification pixel-based approach was used in classifying the image. Water bodies, settlements, rocky land, plain barren area, high vegetation, low vegetation, and agricultural land were classified in the image. The results indicated that malaria occurrences change with the geographical locations, climate, population, distance from the water, and literacy percentage. The outcomes of this study showed that most cases occurred in rural areas. The study also discussed the effect of each factor on the disease. The favorable breeding sites are low vegetation, agricultural land, and water areas. The author suggested that the unhygienic areas and sanitary situation should be improved.

Dengue disease was also researched in Oran City, Argentina, in which the researchers [26] used the dengue cases, temperature, rainfall, humidity, precipitation, Land Surface Temperature (LST), and NDVI for dataset features. They used moderate-resolution imaging spectro-radiometer (MODIS) sensors for data extraction of LST and NDVI. Two linear regression models were used for further processing, i.e., environmental variables with a time lag and without time lags. The author has discussed future work as risk mapping that can be performed in other locations in the northern area of Argentina, such as Tartagal and Salvador Mazza cities.

The authors [27] identified vulnerable areas, helped control them and prevented them by monitoring Zoonotic cutaneous leishmaniasis targeted in the Golestan province in Iran. Visualizing high and low-risk areas is very helpful for public health decision-makers in the geographical management of leishmaniasis incidences and also gives them directions about where their control efforts should be targeted. Temperature, rainfall, humidity, precipitation, evaporation, inverse distance weighting (IDW), NDVI, and DEM have been selected as significant risk factors for further processing. The author used analytical and fuzzy hierarchy process decision-making methods for risk mapping leishmaniasis disease. Some of the limitations in the study were discussed by the authors: for example, data from only three years (2010 to 2012) were used, which is significantly less for achieving good results. Secondly, there is a need to use biological features and socioeconomic data for models’ better performance.

The study [28] measured the effects of environmental and climatic factors such as rainfall, temperature, Normalized Difference Vegetation Index (NDVI), etc., on malaria in Gujrat, Pakistan. Data were analyzed in the form of spatial and temporal patterns. The data processing showed a positive relationship with rainfall, vegetation index, population density, and water bodies and positive and negative relationships with temperature. The results were visualized by GIS software to map areas at high risk of malaria. Moran’s I tool was used to calculate spatial autocorrelation. It was then evaluated that the pattern was random, dispersed, or clustered. This type of research will be helpful for policymakers to control the disease in the area. The author suggested that other factors affecting malaria transmission should also be studied for future research. There is another future recommendation to investigate other vector-borne infectious diseases to help public health and policymakers take preventive steps to eliminate these diseases.

Machine learning methods were used for the GIS-based study of the vector of Zoonotic Cutaneous Leishmaniasis in Golestan province, Iran [29]. The used dataset variables were temperature, precipitation, Land Surface Temperature (LST), NDVI, and Digital Elevation Model (DEM). Logistic Regression, RF, and Support Vector Machines (SVM) were used to classify the presence or absence of disease vectors as data labels. After analyzing their results, the authors recommended the SVM for these types of medical problems. An Early Warning System (EWS) to actively monitor the epidemic areas can be implemented to extend this study for leishmaniasis, tick- and other mosquito-borne diseases.

Temperature, rainfall, humidity, wind speed, and the number of dengue cases were the variables used for the dengue outbreak prediction in Puskesmas in Malang regency [30]. Fuzzy logistic regression was used for outbreak prediction of validation data. The accuracy of this model was higher than that of the Neural Network (NN), Naïve Bayes, and Random Forest (RF). Dengue cases and temperature were the most critical factors in this prediction. Proper sanitation and high awareness of society undoubtedly affect the results of prediction. These factors should be used in prediction. The mosquito population variable should be used because it is also included in the list of those factors that influence outbreak.

The focus of the research on Mexico was on the month-wise prediction of dengue cases for the next year [31]. The dataset included spatial and temporal dengue and dengue fever hemorrhagic cases and air temperature. Various time-series prediction techniques were used. Auto-encoding-based time-series Clustering with Nearest Neighbor, Autoregressive Integrated Moving Average and Vector Autoregression, M5’, Support Vector Regression (SVR), and kNN were the methods used. Regression analysis was used to find the relationship between the independent variable (temperature) and the dependent variable (dengue cases). More effective algorithms for time-series clustering should be explored.

The research [32] has developed a WebGIS platform for dengue risk mapping for the district of Lahore, Pakistan. The website displayed the following features: (1) dengue fever areas with a high threat; (2) highest patient count; (3) real-time spatial and other attributes updates about dengue. The analysis was completed by creating low, moderate, high, and very high ranks. Two maps were created: one for the number of patients in each union council. A second map was created by using other data accessed from various sources. The environmental factors used were spatial and tabular data gained from multiple sources: rainfall, population density, LST, NDVI, and land use/land cover. The risk maps were created using the mentioned factors in the GIS environment using a weighted overlay function. A weighted overlay tool arranged the parameters from highest to lowest priority. The Proactive Response, an Integrated System Management framework, facilitated users to view images and maps on the internet in real time. The results for the poor areas showed that highly populated areas had a high risk due to the unclean condition and unsurfaced roads and water accretion state. The rich areas also had dengue transmission risk because they had swimming pools and lawns in their houses as the dengue larva breeds over clean water. The authors obtained data from the WHO that need more features. Patient name, gender, age, hospital name, and date of admission in the hospital are the attributes. There needs to be more attributes for efficient results. Temporal analysis with confirmed cases will give more accurate results.

The study [33] in Brazil’s Espirito Santo on dengue fever cases time-series prediction used the dataset included dengue fever cases, temperature, precipitation (the number of rainy days in the week), relative humidity, surface-level pressure, and precipitable water. More than historical data for dengue cases was needed in many locations; then, the authors of this study used a novel approach to increase the temporal data. The framework ranks incidence data of adjacent locations around a target by combining metrics based on correlation, spatial distance, and local incidence. For this purpose, RNN, LSTM, and GRU were the models to predict dengue fever cases accurately. The environmental and economic data based on locations by combining the metrics in feature ranks can be used in the future. The proposed model depended on temporal data available at a reasonable spatial resolution.

This study [34] focused on geographical factors affecting malaria and analyzed the other factors that can control malaria transmission. Remote sensing was used to gather data on environmental factors affecting malaria in Ethiopia’s two different geographical locations. Malaria varied with the geospatial factors, including temperature, precipitation, vegetation, land use, surface water, NDVI, Normalized Difference Moisture Index (NDMI), and LST, because different geographical locations have variations in the environmental factors. This study analyzed the relationship between malaria incidences and environmental data by Boosted Regression Tree (BRT) models. The sub-districts with less greenery and moist air in the malaria spread season had the most significant number of cases. This research has a limited scope to classify settlements through PlanetScope imagery because a very high-resolution dataset will give more accurate results.

The factors used in the research [35] conducted in northeastern Thailand on the spatial distribution and prediction of abundance (classified as high and low) dengue vector were household-based socioeconomic and urban–rural residence, education status, household income, socioeconomic status, dengue knowledge, attitude and practices, and land use/land cover (built-up area, permanent wetlands, natural tree cover, rubber plantation, rice crop). The authors compared the results of all these different types of data factors and also used machine learning models on all these different data factors collectively: Logistic Regression, SVM, RF, k-Nearest Neighbor (kNN), and Artificial Neural Network (ANN). RF achieves the best model performance with all the data variables used in this study, but temperature, rainfall, and humidity were not used in this study and should be used with increasing data.

The authors [36] categorized the leishmaniasis species spatially, and they mapped the five types of diseases in the form of risk patterns depending on climatic factors in French Guiana. The climate factors, temperature, and rainfall influence the leishmaniasis species. The tree-cutting loss also has a significant impact. The study discussed the effects of temperature, rainfall, humidity, and deforestation on different leishmaniasis. Stacked Species Distribution Models (SSDMs) effectively find the high-risk regions of leishmaniasis species. Further research could use SSDMs to analyze the spatial patterns of leishmaniasis species. Studies can further work on the distribution of leishmaniasis species according to seasons.

The geographically weighted Poisson regression (GWPR), generalized linear models (GLMs), Ordinary Least Square (OLS), and QGIS were used in the study [37] conducted in the Buhera rural district, Zimbabwe for mapping malaria hotspots to identify the contribution of environmental factors in malaria spread. The data features extracted from some satellites MODIS and CHIRPS were malaria cases, rainfall and calculated NDVI. Enhanced vegetation index (EVI), LST (day and night), socioeconomical, biological, and environmental factors such as altitude, humidity, and Normalized Difference Water Index (NDWI) are also important factors in malaria occurrence that should be used in the future.

These studies consisted of data features related to climate, socioeconomic factors, environmental factors, disease incidences, etc., but we observed climatic features, so the count of each climate factor is mentioned here; the temperature was used in 12 studies, while humidity was used in 8 studies, precipitation in 5 studies, rain in 10 studies, and LST in 5 studies. From these studies, temperature, humidity, and rain are the most used risk factors. Mostly fuzzy logic methods and RF were used in studies for predicting disease. Neural networks, SVM, kNN, and Logistic Regression were the models used by two studies; the remaining models were used by only 1 study each. Some papers used novel approaches such as hybrid models. Some studies were comparison-based.

## 3. Methodology

### 3.1. Workflow

**Figure 2:**
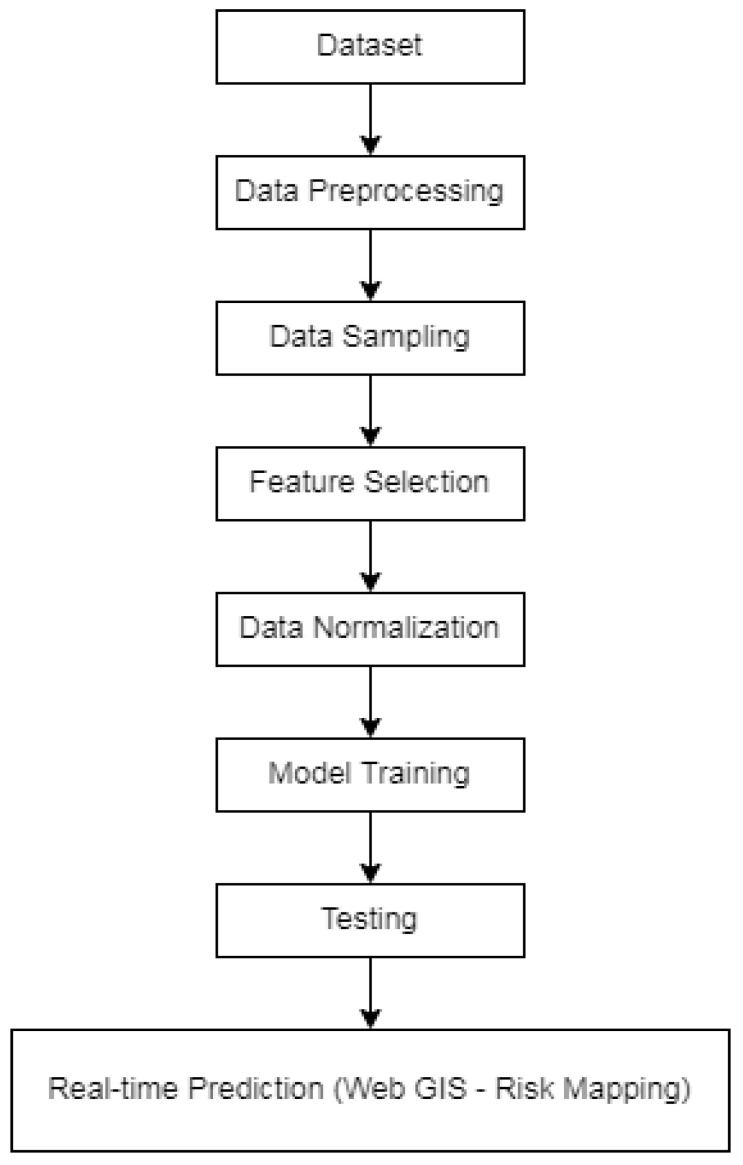
Workflow of proposed method of data processing and machine learning model.

The step-by-step procedure of the proposed methodology is discussed here. The workflow for our methodology is shown in Figure 2.

In the first step, we have a supervised dataset of a size of 59,662 rows and 23 columns. The data consists of features from patients’ cases, climate, and socioeconomic and population data. Initially, the data were in raw form, including missing values and outliers, so it needed to be processed before analyzing the data and generating results. The data preprocessing step was the second step. Next, 4 data samples of different sizes were created. A feature selection process has been used on these data samples to analyze the performance of climatic features, i.e., precipitation, temperature, and specific humidity. Then, standard scalarization was used for data normalization to remove outliers in the dataset. Five different machine learning classifiers were used to train and validate data. To identify the model’s efficiency, the testing phase was also conducted on unseen data. The testing data randomly selected 20% of all data before training. The real-time prediction was made to map the risk locations on developed WebGIS.

### 3.2. Study Area

**Figure 3:**
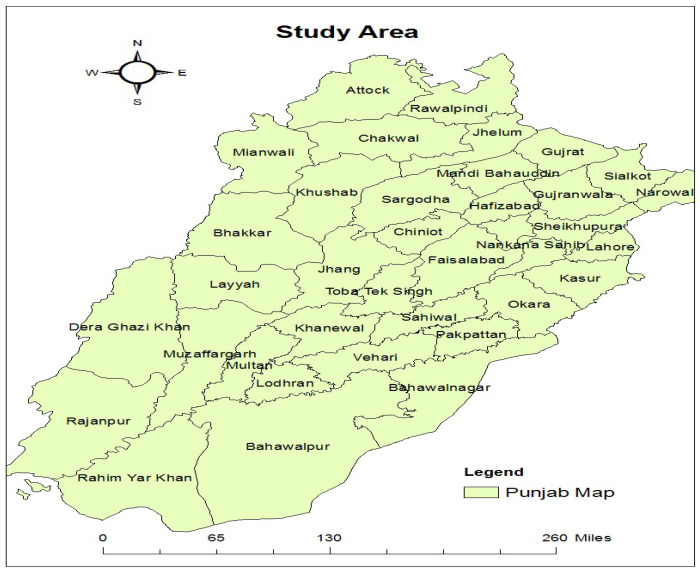
Geographical representation of Punjab.

This study focuses on dengue, malaria, and leishmaniasis in Punjab, Pakistan. Punjab is a province of Pakistan. It has a population of approximately 110 million, according to the 2017 Pakistan Census [38]. It is the most populous province and second largest province by area after Balochistan. Punjab is located at 30°2′42.8856″ N and 72°20′55.9284″ E latitude and longitude, respectively. Its total area is 205,344 km^2^ [38]. The province is divided into nine divisions, 36 districts, and 145 tehsils [39]. Lahore is the capital and largest city of Punjab. From west to east, five rivers flow through Pakistan’s Punjab province. Punjab’s region temperature ranges from −2 to 45 °C; in winter, the temperature can go down to −10 °C, and in summer, it can rise to 50 °C. Figure 3 shows the Punjab map. The Punjab region has three main seasons: (i) hot weather season from April to June when the temperature rises to 51 °C; (ii) the rainy season from July to September; and (iii) cold weather from October to March, and the temperature goes down to 2 °C [38]. Figure 3 was created on a tool named ArcGIS by using a shapefile of Pakistan with districts’ boundaries; then, we created a new layer for the province of Punjab with districts’ boundaries from the previous layer. ArcGIS is a powerful analysis tool that can perform the most fundamental GIS operations.

### 3.3. Dataset

The dataset was available in Excel format. The time duration of the data is 2014 –2018. We obtained the dataset from another researcher (a student from our university) who extracted climate features from satellites and patient data gathered from the Disease Surveillance System (DSS) of the Punjab IT board. The dataset contains 23 features with 59,662 records. These features are related to patients’ cases, climate, socioeconomic and population data. These features are listed in Table 1.

#### 3.3.1. Patient Data

**Figure 4:**
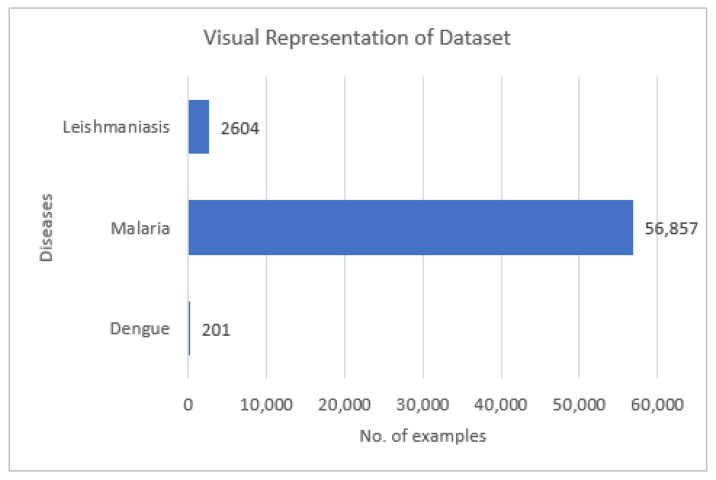
Visualization of total no. of examples of each disease in dataset.

The patients’ data consist of the disease diagnosed, patient, district, Rural Health Clinic (RHC)/Name, the latitude and longitude of that RHC, and the date/time of that patient’s entry into the system. Diseases include dengue, malaria, and leishmaniasis. Districts included are from Punjab province only. The patient data were used to indicate the number of cases for each disease. Diseases were used as labels for our dataset in the prediction models. The total number of patients for each disease is visualized in Figure 4. In total, 2604 cases of leishmaniasis, 56,857 recorded cases of malaria, and 201 total cases of dengue were included in our dataset.

#### 3.3.2. Climate Data

The features from climate data included in our dataset are precipitation, temperature, and specific humidity. Values of temperature are in kelvin. Precipitation is in mm/h. Humidity is the ratio of the mass of actual water vapor to the mass of vapor in moist air. The temporal and spatial changes in climate factors such as precipitation, temperature, and humidity affect vectors’ ecology and biology and result in a risk of disease transmission. The survival and development of vectors of VBDs are reliant on climate features [40].

#### 3.3.3. Socioeconomic Data

The total number of features used for socioeconomic data is 13. Most features are regarding the water, i.e., people have access to pure water or how they treat water before drinking, etc. Stagnant and dirty water is the primary cause of malaria. Dirty puddles and dirty water are high mosquito breeding places and are some of the reasons for malaria [41]. The reasons for poverty are less availability of health facilities and the environmental conditions of people’ s living and working areas. Some specific groups of the population are at risk for leishmaniasis. In rural and peri-urban areas, houses are built with poor-quality materials (mud, straw, bamboo), which can become the reason for sandflies’ shelter and can cause leishmaniasis. A lack of sewage and garbage collection services, leading to the accumulation of waste, can also become the reason for vector breeding places [42]. Socioeconomic factors have an impact also on dengue. The dengue epidemic remains due to socioeconomic factors [43].

#### 3.3.4. Population Data

The population for districts shown in the study area in the shape of the polygons in Figure 3 was included as a feature in the dataset named Population [Population Census 2017—Provisional Results] as mentioned in Table 1. Population data are linked with socioeconomic features. Population data were considered for each district, and socioeconomic features were also considered for each district.

### 3.4. Data Preprocessing

There is always a need to clean the data before training a machine learning model. The technique we used in preprocessing steps was removing unnecessary columns. The second was used to fill in the missing values or NA values. The missing values were filled by an interpolate method in the python programming language. We used the column-wise linear interpolation method to fill in the missing values. Linear interpolation fills the value ranges between, above, and below rows of known values in a column. After this step, the features that remained in the dataset were 18 out of 24.

### 3.5. Data Sampling

**Figure 5:**
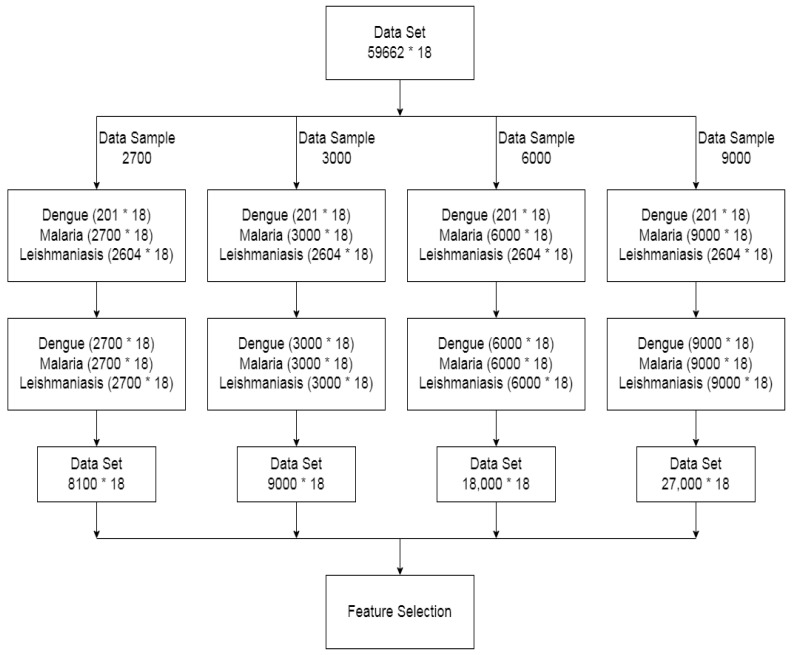
Synthetic minority oversampling technique (SMOTE) on data samples.

This is very important for the good performance of a machine learning model to have a dataset with balanced classes. The class imbalance between majority and minority classes can bias the predictive performance of machine learning algorithms. Our dataset has 3 imbalanced classes: 201 records of dengue, 56,857 records of malaria, and 2604 records of leishmaniasis then 18 is the no. of columns in each data sample as shown in figure (no. of rows * no. of columns). To solve this issue, we used the synthetic minority oversampling technique (SMOTE). It balances the class distribution by randomly increasing the minority class examples by replicating them. SMOTE analysis was performed on the four sizes of malaria (majority class) category data to oversample minority classes, i.e., dengue and leishmaniasis. The four data samples were created, i.e., 2700, 3000, 6000, and 9000. Each class was oversampled to become equal to the malaria class, and we used four different sizes of the malaria class to oversample dengue and leishmaniasis to these four different sizes. We denoted these data samples with the size of the malaria class. Then, concatenating these three classes, the total size of these four datasets becomes 8100, 9000, 18,000, and 27,000, as shown in Figure 5.

### 3.6. Feature Selection

Feature selection has been made based on climatic features. We wanted to analyze which climate feature is important for these diseases. For this purpose, we used manual feature selection. We excluded all climate features; only temperature was included in the dataset and then, we generated the results. Next, we again follow the same procedure for other climate features. Only one climatic feature was included each time for every above-discussed data sample. In addition, we used all features of climate (temperature, precipitation, and specific humidity) data together with other features of the dataset for comparison.

**Figure 6:**
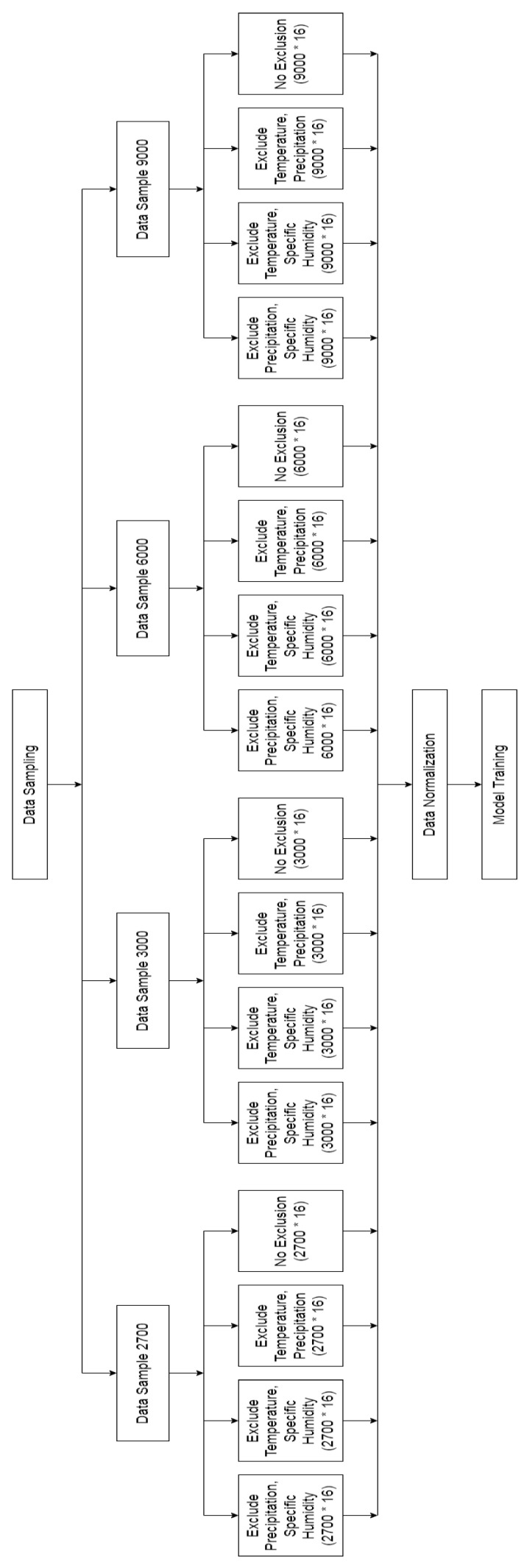
In-depth flow diagram of manual feature selection.

Figure 6 shows the complete diagram of the feature selection phase. It represents all four data samples. Each sample data included each climate factor in every iteration with other features. The remaining two features from climate data were excluded for every iteration. Then, all data were used for data normalization and model training. We analyzed all these datasets and selected the best dataset after the model training phase. Each dataset contained a different shape (no. of rows * no. of columns), as also mentioned in Figure 6.

### 3.7. Data Normalization

The dataset was collected from different satellite images containing noise and outliers. Before using data for training the model, we must use any method to remove these outliers. Outliers are the values of a dataset’s features with large differences between ranges. There are many techniques to resolve this issue. The Z-score normalization has been used for this task. The standardization or Z-score normalization puts all features on the same scale. It measures the standard deviations below or above the mean.

(1) $$Z=\frac{x-\mu }{\sigma }$$

*Z* is the standard score. X is the observed value in a feature in each iteration. μ is the mean of the training samples. σ is the standard deviation. We used the built-in function StandardScalar() for z-score normalization in python. It uses the formula in Equation (1) [44].

### 3.8. Prediction Models

The machine learning algorithms are used to perform VBDs risk prediction. The selected model was used to predict risk maps on WebGIS for real-time prediction. Five different machine learning models were used in our research. The models were Light Gradient Boosting Machine (LGBM), Random Forest (RF), Decision Tree (DT), Support Vector Machines (SVM), and Multi-Layer Perceptron (MLP). The models were trained using sklearn documentation for RF, DT, SVM, MLP, and LightGBM documentation for the LGBM model in python [45,46]. Supervised machine learning built-in functions can be used easily but should require tuning some hyperparameters for this purpose. The user should set the value of hyperparameters before running the code or using default values from the software package. The reason for this step is to obtain the optimal predictive performance of a model [47]. Accuracy, precision, recall, and f1-score were calculated to analyze the algorithms’ performance and select the best algorithm [48]. To identify whether our model is an underfit or overfit model, we created learning curves on accuracy for training and validation data using k-fold cross-validation to train and test data. The learning curve on a negative mean square error on data was generated. The detailed models’ descriptions are also added below.

#### 3.8.1. Light Gradient Boosting Machine (LightGBM)

LightGBM is short for Light Gradient Boosting Machine. LightGBM is a gradient-boosting technique based on the decision tree algorithm suggested by Microsoft research in 2017. LightGBM grows trees using a leaf-wise approach. This approach is also called best-first. The leaf-wise approach achieves less loss than the level-based (depth-wise) grow approach, which is mainly used by decision tree algorithms. The LightGBM uses a leaf-wise grow method with maximum depth limitation to avoid overfitting. It splits the leaf node with the highest gain at each iteration. It requires less memory usage because only one leaf node splits each time. This process consumes less computation and trains the model fast and efficiently [46,49].

LightGBM is a type of gradient-boosting model. The max depth of a tree was limited by mentioning it explicitly. Gradient Boosting Decision Tree was used as a gradient boosting type for this model to use the combined effect of the weak learners to achieve the strong learner. In this model, weak learners are decision trees, and each tree is used to reduce the error of prior trees [50].

#### 3.8.2. Random Forest (RF)

The Random Forest (RF) classifier contains multiple decision trees on different sub-samples of the dataset. It does not create a decision tree to make an optimal segmentation point, but sub-optimal segmentation is performed using randomness. It uses average predictive accuracy due to its usage of multiple decision trees on different subsets of datasets and control overfitting. It can create a highly accurate classifier using a large number of features [51,52]. RF is an ensemble technique of n untrained decision trees. Each tree consists of a root node with a data sub-sample. These sub-samples are generated from our labeled training data by randomly choosing m<M features, where m is much smaller than M, which is the total number of features in our data. n sub-samples are called bootstrap samples. It takes the majority vote from the predicted classes. It can handle a large number of features without deletion. The important variables in classification can also be assessed [53].

The RF classifier has hyperparameters to be initially set by a user before calling its function in a code. Tuning these hyperparameters improves the performance of the model. Some of these parameters are the number of examples randomly selected for each tree, the total number of trees used to train the model, the number of features used for each split, etc. [54]. We used n estimators, the maximum number of trees used before the majority voting, and max depth, which means each tree is allowed the total number of splits until it reaches the max depth, and other hyperparameters remained the default. The values of these hyperparameters are chosen by the hit and trial method.

#### 3.8.3. Decision Tree (DT)

A Decision Tree (DT) is a classifier that works as a recursive partitioning. Each internal node in the tree splits into one or more sub-spaces. Each leaf node represents a class. Each branch of the tree illustrates the possible decision scenarios and their results [55]. The root node contains all the training data. The greatest separation value, X, splits the nodes. For the next split, if X is less than the threshold, send the data to the left side; otherwise, send the data to the right side of the node. Then, the same process repeats. Our dataset features contained numerical values. CART has no restriction for the features to be categorical or numerical in classification problems, and scikit-learn also provides CART implementation. CART uses thresholds and features, calculates the maximum information gain on one node in each iteration, and builds binary trees [56].

We used all the hyperparameters as the default except for the max depth, which is the maximum depth of a tree that the model will not reach because of the disadvantages, and overfitting is one of them. The maximum depth a user can give to this model is one less than the total number of examples [57].

#### 3.8.4. Support Vector Machines (SVM)

SVM is widely used to solve classification problems. SVM is efficient in handling non-linear data. It converts non-linear classification data into a high dimension to make it linear. It finds the hyperplane that separates the classes. The hyperplane maximizes the margin between classes [51]. The closest point of each class to the separated hyperplane is called the support vector.

Picking an optimal value for hyperparameters is a significant step for SVM. These values can be chosen by reducing errors and increasing the model’s performance. The kernel performs the mapping of non-linear data points. We used the radial basis function as the kernel in this model [58].

#### 3.8.5. Multi-Layer Perceptron (MLP)

Multi-Layer Perceptron (MLP) is a feed-forward neural network. It is a directed graph of multiple layers. After the input layer, each layer is fully connected with the next layer. It trains the model with backpropagation. MLP is easy to implement but has complex calculations. That is why MLP trains the model slowly [59].

Hyperparameters regarding the initialization and updating of weights were used. The solver function adam was used for iterative weights update. It is a stochastic gradient-based optimizer. Ten hidden layers were used on a hit-and-trial basis. The distinct number of neurons for these layers was used in this study.

### 3.9. Evaluation Metrics

We used the evaluation metrics to test machine learning models: precision, recall, f1-score, accuracy, confusion matrix, and learning curves for training, validation, testing data, and calculated error.

*Accuracy* represented in Equation (2) illustrates the fraction of the total no. of examples correctly predicted with the total no. of examples.

(2) $$Accuracy=\frac{Tp+Tn}{Tp+Fp+Tn+Fn}$$

*Tp, Fp, Tn, Fn* are the true positive, false positive, true negative, and false negative values, respectively.

The *recall* is calculated as the fraction of no. of samples and predicted as a positive class with the total no. of samples positive. It is also called the true positive rate. Equation (3) represents the formula of recall.

(3) $$Recall=\frac{Tp}{Tp+Fn}$$

*Precision* is calculated as mentioned in Equation (4). It is also known as the positive predictive value. It is the formulation of no. of samples predicted as positive from the total number of samples predicted as positive.

(4) $$Precision=\frac{Tp}{Tp+Fp}$$

The f1−score is also called the harmonic mean of precision and recall and is mentioned in Equation (5).

(5) $$f1-score=\frac{2\times Recall\times Precision}{Recall+Precision}$$

Equation (6) shows the simplified form of f1−score .

(6) $$f1-score=\frac{2\times Tp}{2\times Tp+Fp+Fn}$$

The confusion matrix demonstrates the classification of a model. The confusion matrix represents the no. of examples predicted in each class. It is in the form of a matrix. We have three classes. A 33 *x* matrix is calculated as a confusion matrix [60].

### 3.10. Website Development

**Figure 7:**
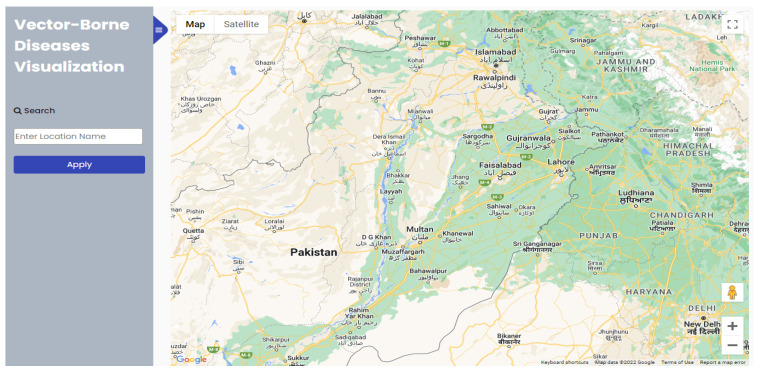
Website User Interface centering Punjab Pakistan Map—Home Page.

We have created the website using GIS for real-time risk mapping and visualization of dengue, malaria, and leishmaniasis based on generated results from a trained machine learning model. It is developed using the python framework Django, HTML, CSS, Bootstrap, JavaScript, jQuery, Google Map API, and OpenWeatherMap API. The GUI of the website is shown in Figure 7. The website contains on the left side a side menu. This side menu consists of an input form to enter any district name from Punjab; then, we click the ‘Apply’ button in the user search area to see how the website responds to risk in the searched district. A search bar to search a district is located on the side menu under the website’s title (Vector-Borne Diseases Visualization). After searching, the district machine learning model predicts diseases from three vector-borne diseases (malaria, dengue, and leishmaniasis) and plots them on the map in specific colors for each disease.

The remaining screen area represents the World Map pointing toward the coordinates of Punjab, Pakistan. To use the world map and visualize it, we used Google Maps. The Google Maps platform provides Maps JavaScript API. It is for the developers to use the maps in their own created websites and mobile apps. It contains four basic map types: terrain, roadmap, satellite, and hybrid [61]. We used a roadmap as a type of map for our website. To represent the locations on Google Maps, we used the latitude and longitude of the Union Councils of Punjab. The latitude and longitude for union councils are created from ArcGIS using the Pakistan union councils’ shapefile. Then, we extracted the latitude and longitude of Punjab in an Excel file.

Various visualization layers are used to customize Google Maps to visualize the data in different formats and shapes. We used a heatmaps visualization layer to customize Google Maps. Heatmaps make it easy for users to understand the distribution of risk maps. It is used to show the intensity of data; for example, higher-risk areas are plotted more prominently in the heatmaps [62].

OpenWeatherMap API is used to obtain the current weather map. This API collects data from radars, weather satellites and weather stations, and remote sensing satellites, from which one satellite is NOAA [63]. When a user searches a district, the backend code finds the latitude and longitude for that district’s union councils and then sends requests with that latitude and longitude to API to obtain the current weather for each union council. We extracted temperature, precipitation, and humidity from that data.

**Figure 8:**
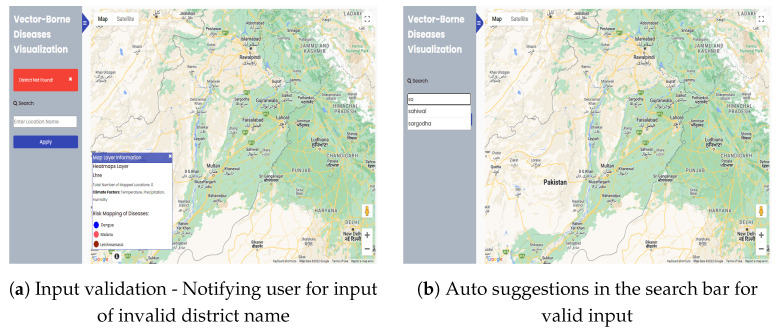
Additional features for valid input location.

The functionality of validating input is also used in this website so that accurate results are provided to users. If a user enters the wrong input in the search bar to search a district, i.e., wrong spelling or any other string or district from other than Punjab, Pakistan, then an error ’District Not Found!’ is displayed as shown in Figure 8a. Users can correct this error by writing the correct district name or by using the autosuggestions, which gives a list of names of districts, as shown in Figure 8b.

### 3.11. Real-Time Risk Prediction

**Figure 9:**
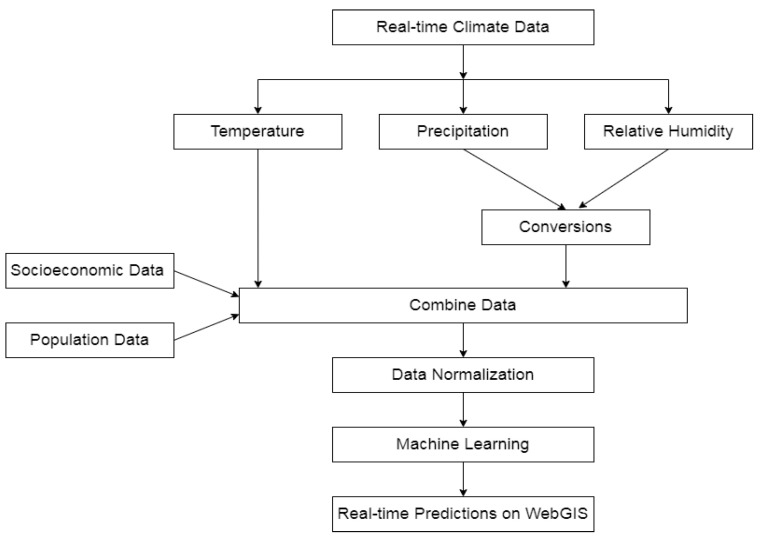
Workflow of real-time predictions on WebGIS.

The user can enter the input after the website loads on a browser. We used the results of the historical data that had been used in training to predict new risk maps for the user-searched district. The predictions were created for each union council of that district. The predicted risk locations were plotted on Google Map API. All this process can be summed up in these steps: input, obtaining current climate data, conversions of climate features in units according to training data, machine learning processing, generating risk locations, loading the website, and plotting risk maps. The workflow of real-time predictions is shown in Figure 9.

We saved a machine learning training model in a file, and then, it was used in the backend code of the website. The backend work has been completed in the python programming language, where OpenWeatherMap API has been used to access real-time climate data from satellites and weather stations. After obtaining the climate data, the conversion of values of each variable in previously used units must be achieved. The temperature was in kelvin, so there was no need to convert it. Precipitation was in mm for the current minute; we must convert it to mm/h so that the precipitation value would be multiplied by 60. Humidity was in %, which is relative humidity [64]. Relative humidity must be converted to specific humidity before use. This takes some equations to calculate specific humidity using relative humidity, temperature, and pressure.

(7) $$e=6.11\times 10^{\frac{7.5T_{d}}{237.3+T_{d}}}$$

(8) $$e_{s}=6.11\times 10^{\frac{7.5T}{237.3+T}}$$

*e* calculated in Equation (7) is the actual vapor pressure and $e_{s}$ calculated in Equation (8) is the saturated vapor pressure. *T* is the air temperature. $T_{d}$ is the dew point temperature.

(9) $$RH=\frac{e}{e_{s}}$$

(10) $$w=\frac{eR_{d}}{R_{v}\left(p-e\right)}$$

To verify relative humidity, we used Equation (9). Next, we calculated *w* in Equation (10) to calculate specific humidity. *w* is the mass mixing ratio of water vapor to dry air. $R_{d}$ is a specific gas constant for dry air in unit J kg^−1^ K^−1^. $R_{v}$ is a specific gas constant for water vapor in unit J kg^−1^ K^−1^.

(11) $$SH=\frac{w}{w+1}$$

*SH* calculated in Equation (11) is the specific humidity or ratio of water vapor mass to the total mass of air and water vapor. It is calculated using relative humidity, temperature, and pressure [65].

The prediction step using a trained model has been completed. In the next step, google map API JavaScript was used to connect the back-end results with the front-end by latitude and longitude, and then HTML, CSS, and Bootstrap were used to visualize the website on a browser to display our results to the user.

## 4. Results and Discussion

### 4.1. Dataset Description

**Figure 10:**
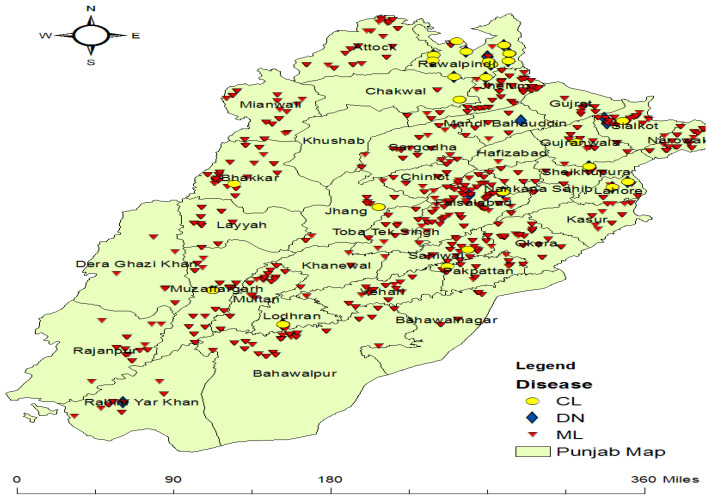
Visualization of reported cases in Punjab for malaria, dengue and leishmaniasis.

The visualization of reported cases from the study area (Punjab) for dengue, malaria, and leishmaniasis can be seen in Figure 10. Different shapes were used to differentiate between these three diseases. Yellow circles, blue diamonds, and red triangles represent cutaneous leishmaniasis, dengue, and malaria. The red color is used to indicate the high number of malaria cases, the yellow color signifies the moderate number of leishmaniasis cases, and the blue color is for a smaller number of cases of dengue in Punjab. This map was created with a tool named ArcGIS. These cases were plotted by their latitude and longitude from the dataset in the Excel sheet, where each patient record is represented with latitude and longitude to indicate the reported case’s geographical location.

#### 4.1.1. Dengue Cases 2014–2018

**Figure 11:**
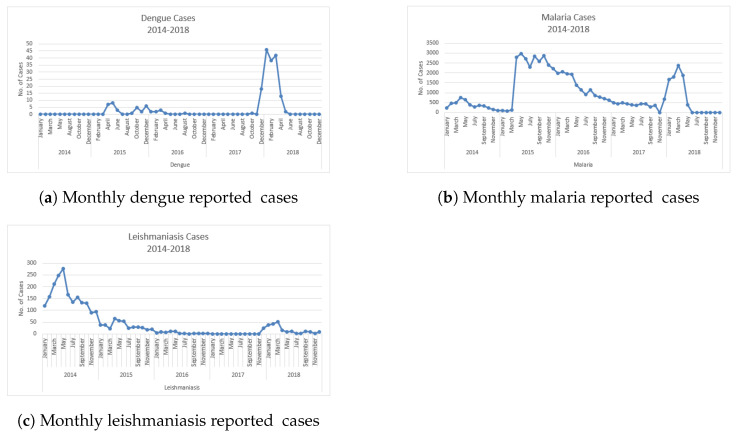
Monthly reported cases of each disease from 2014 to 2018.

Dengue cases from 2014 to 2018 are shown in Figure 11a. The line graph shows the no. of cases in each month. The maximum no. of cases reached 46 in January 2018; then, it decreased to 38 in February and again increased in March to 42. With the changes in climate in April 2018, dengue incidences remained at only three and then two in May 2018. Mostly dengue spreads in the winter season.

#### 4.1.2. Malaria Cases from 2014–2018

The visualization of malaria cases in Punjab, Pakistan, from 2014 to 2018 is shown in Figure 11b with a line graph. The maximum no. of cases reached 2960 in May 2015. After a large no. of cases of malaria, it gradually decreased in 2016 and 2017; then, it increased again in December 2017, reaching 682 and stopping at 2383 cases in March 2018. Malaria mostly remained at its peak from April to June (hot season).

#### 4.1.3. Leishmaniasis Cases from 2014–2018

The leishmaniasis case data are shown in Figure 11c. Leishmaniasis cases reached 276 in May 2014. This was the highest number of cases in the history of leishmaniasis until 2018. Leishmaniasis cases from 2016, 2017, and 2018 were less than 51. Once in 2018, March cases reached 51, but after that, cases decreased to fewer than 17. Mainly, this disease spread from December to June.

### 4.2. Relationship of Climate Factors with Vector Borne Diseases

We used the spatial interpolation method IDW in ArcGIS to find the unknown values of the climatic factors for each disease. The below images visualize the results of the findings of this technique. Using this method, we found the abundance maps used in previous studies [66]. We used these maps to link each climate factor with each disease. All these results were generated using ArcGIS. The Punjab map and our dataset of 59,662 records which included each climatic feature for each disease were used to generate interpolation results. Nine classes were generated with an equal interval between values. These classes represented values from low to high for temperature, precipitation, and specific humidity for dengue, malaria, and leishmaniasis. We can quickly identify the minimum and maximum values of each climatic factor for each disease. We chose different colors for each class, from low to high intensity, to better visualize the results.

#### 4.2.1. Relationship of Temperature with Each Disease

**Figure 12:**
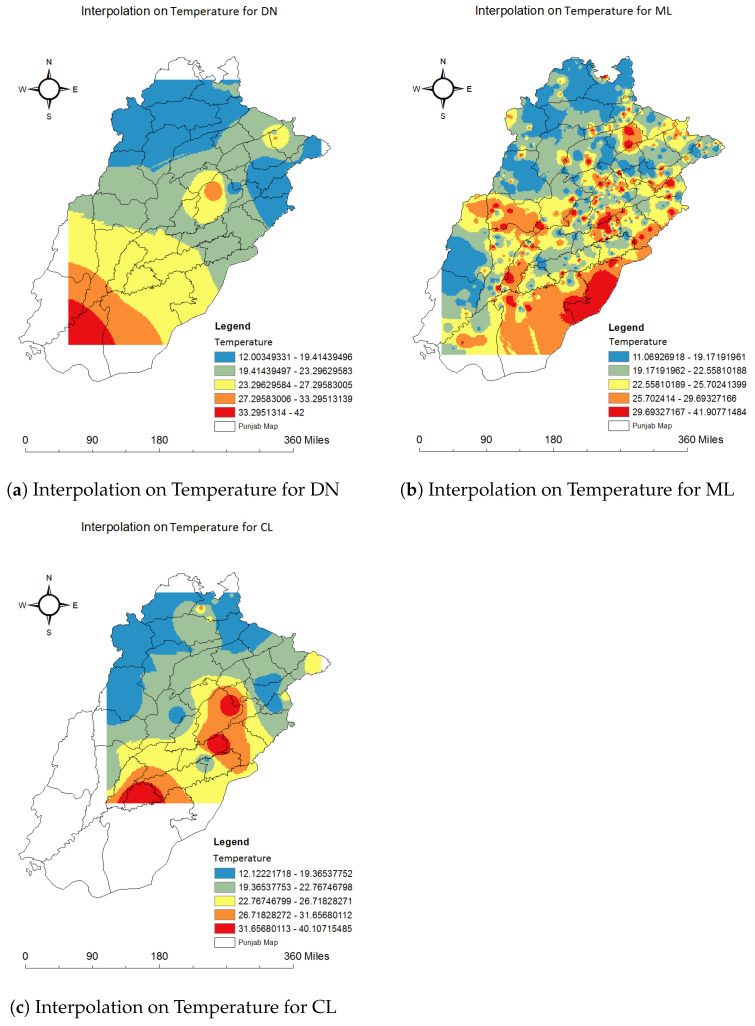
Relationship of temperature with dengue, malaria and leishmaniasis using interpolation.

Temperature ranges from 12.003 to 42 °C for dengue (DN) disease, as shown in Figure 12a. The map of Punjab in the figure shows that most of the cases occurred in places where the temperature is from 12.003 to 27.296 °C.

The relationship of temperature with the disease malaria (ML) is represented in Figure 12b. The minimum temperature value is 11.069 °C, and the maximum temperature is 41.908 °C. These values are represented in various categories, from low to high in blue to red. The red color shows the highest created range scale of temperature for malaria. These results show that malaria is a disease affected mainly by all ranges of temperature values.

The relationship of temperature with cutaneous leishmaniasis (CL) can be seen in Figure 12c. The lowest temperature for leishmaniasis cases is 12.122 °C, and the maximum temperature value is 40.17 °C. The categories for high-temperature values from 26.718 to 40.107 °C contain fewer cases. It can easily be seen that leishmaniasis is a disease that can spread in low temperatures mostly.

#### 4.2.2. Relationship of Precipitation with Each Disease

**Figure 13:**
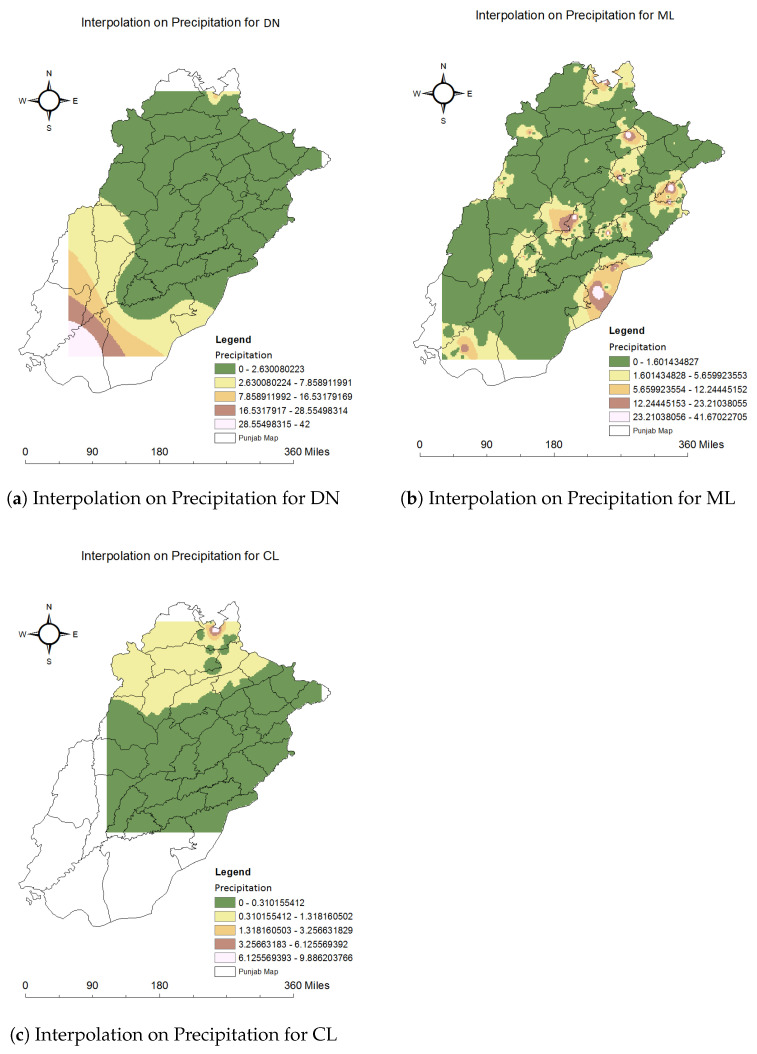
Relationship of precipitation with dengue, malaria and leishmaniasis using interpolation.

The precipitation values range from 0 to 42 in our dataset, showing dengue cases in different Punjab districts. Significantly, a lower effect of precipitation on dengue is visible in Figure 13a. First, Category 0 to 2.63 is shown in dark green color for the low intensity of the precipitation.

Malaria in Punjab is shown in Figure 13b with its minimum and maximum range of values from 0 to 41.67. This minimum to maximum range of values is categorized into five classes. Mostly, malaria cases are in those areas where the precipitation is low; that area is filled with dark green. Moderate precipitation values in some parts of various districts, represented in light yellow, can be seen in Figure 13b.

Rawalpindi contained some parts with high precipitation values with leishmaniasis cases, but most of the parts of Rawalpindi also had less precipitation. Less precipitation is represented with dark green color. Categories created with values from 0 to 9.886 of precipitation existed in Punjab for leishmaniasis cases. All these results are plotted on the Punjab map in Figure 13c.

#### 4.2.3. Relationship of Specific Humidity with Each Disease

**Figure 14:**
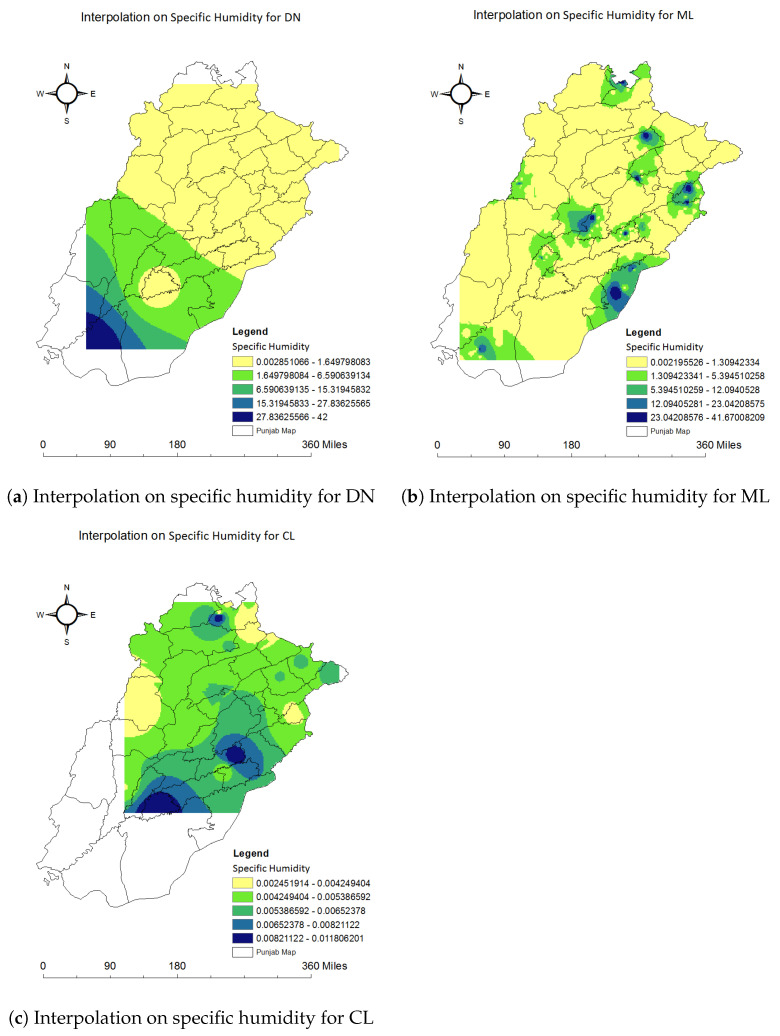
Relationship of specific humidity with dengue, malaria and leishmaniasis using interpolation.

Interpolation on specific humidity for DN is plotted on the Punjab map in Figure 14a. All the dengue cases occurred between 0.003 and 42 range of specific humidity values. We generated various humidity classes to see their impact on dengue mosquitoes. Here, 0.003 to 1.650 is our dataset’s first and lowest category of specific humidity for dengue cases. Very few cases occurred in the category of highest values, 27.836 to 42.

Interpolation on specific humidity for ML is represented in Figure 14b. The lowest humidity for malaria cases is 0.002 to 1.309. The highest humidity is 23.042 to 41.67. The categories from lowest to highest are shown in Figure 14b from yellow to blue colors. Most of the malaria cases occurred in the humidity from 0.002 to 1.309.

The lowest specific humidity for CL is 0.002 to 0.004. The highest value for specific humidity for CL is 0.008 to 0.012. Values from almost all the categories were recorded in different districts during the time leishmaniasis cases were reported. Categories are shown in the figure with the color scale from yellow to blue. Interpolation on specific humidity for CL is shown in Figure 14c.

### 4.3. Results of Machine Learning Models with each Data Sample

The results of testing data after training the model were calculated as a classification report. The classification report shows the precision, recall, f1-score, and accuracy results for dengue, malaria, and leishmaniasis. Table 2 represents these results. These results were in the form of precision, recall, f1-score, and accuracy percentages. We must select the best training model from all four data samples.

**Figure 15:**
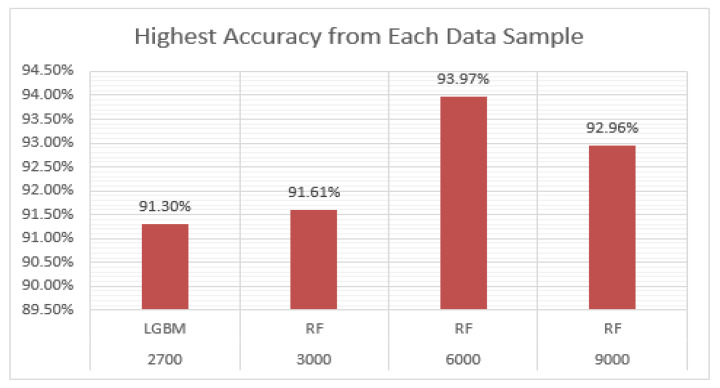
Highest accuracy achieved from each data sample.

The highest accuracy achieved from each data sample is displayed in the graph shown in Figure 15. All the highest accuracies were obtained from a combination of all climate features, socioeconomic, population, and patients’ cases datasets. LGBM achieved 91.30% accuracy on a data sample of 3000. RF achieved the highest accuracies from all remaining data samples. These results show that temperature, precipitation, and specific humidity have a combined effect on dengue, malaria, and leishmaniasis. The results showed that LGBM is an efficient model for small datasets, and RF is the best model for large datasets.

**Figure 16:**
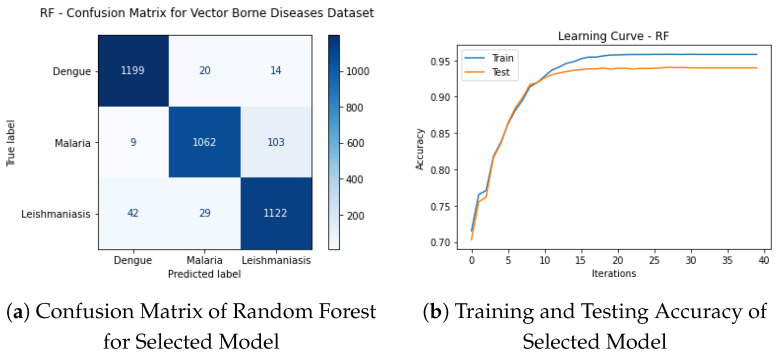

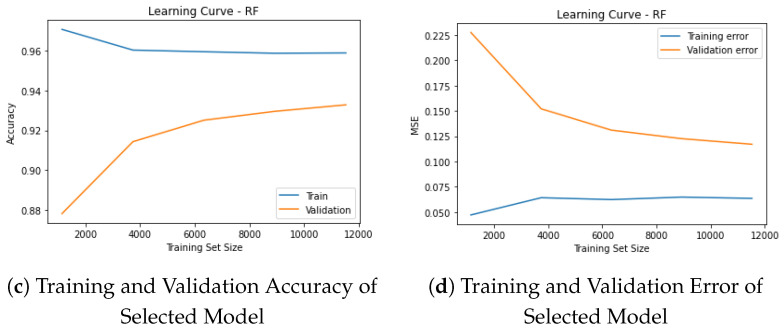
Results of Selected Model.

The maximum accuracy from Random Forest from a data sample of 6000, including all features, is 93.97%. Figure 16a represents the confusion matrix for this model.

The learning curves for the training and testing data accuracy of selected model are shown in Figure 16b. The accuracy achieved for testing data is 93.97%.

We used k = 5 in k-fold cross-validation. The final accuracy for fifth-fold on training data was 95.94%, and on validation data was 92.99%. Figure 16c shows the learning curve of training and validation accuracy of selected model.

The training and validation data error of the selected model is shown in Figure 16d. The lowest error achieved for validation data is 0.125.

### 4.4. Real-Time Model Prediction on WebGIS

**Figure 17:**
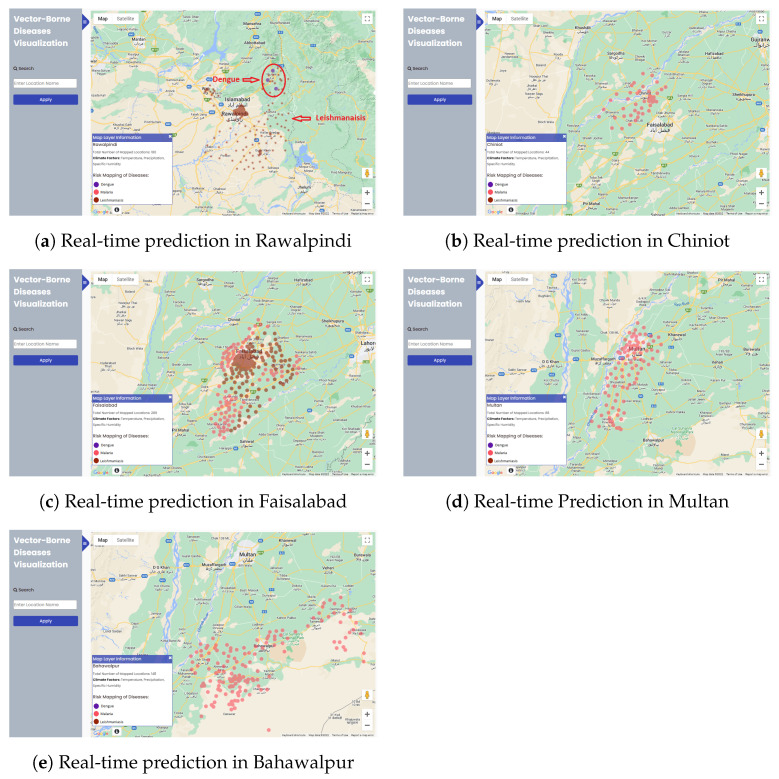
Real-time prediction of various cities of Punjab on WebGIS.

Figure 17a showed that three locations were at risk from dengue represented with small filled purple color circles. The remaining mapped 177 out of 180 indicated the risk locations of leishmaniasis in Rawalpindi in brown tiny circles. Dengue risk locations occurred near Murree. We labeled both diseases and encircled the vulnerable dengue areas in the figure with red color. Real-time dengue and leishmaniasis risk prediction verify the data shown in Figure 11a,c respectively. The following are the minimum and maximum values of climate variables for both diseases in the Rawalpindi district: dengue: temperature: 300.02–300.38 (26.87 °C–27.23 °C), precipitation: 0, specific humidity: 0.0062–0.0067 and leishmaniasis: temperature: 296.26–309.13 (23.113 °C–35.98 °C), precipitation: 0, specific humidity: 0.0053–0.0085.

Real-time prediction in Chiniot is displayed in Figure 17b. The pink color was used to denote Malaria. Chiniot was covered with Malaria in all union councils. The temperature, precipitation, and humidity were favorable for the breeding sites of Malaria in June 2022. The results can be verified by Figure 11b. The total no. of mapped union councils for the Chiniot district was 44. Climate features contained the following values: temperature: 309.69–310.56 K (36.54–37.41 °C), precipitation: 0, specific humidity: 0.0055–0.006.

The results were generated in June 2022 in the Faisalabad district. Some union councils contained the risk of leishmaniasis, and some had the risk of Malaria. In Figure 17c, pink and brown colors represent malaria and leishmaniasis, respectively. The total mapped locations for the Faisalabad district were 289. The total risk mapped locations of malaria were 89 and 200 locations for leishmaniasis. Results of malaria and leishmaniasis can be verified by Figure 11b,c respectively. Values of climate feature for malaria were temperature: 314.53–315.3 (41.38 °C–42.15 °C), precipitation: 0, specific humidity: 0.006–0.0065, and for leishmaniasis were temperature: 314.59–315.1 K (41.44–41.95 °C), precipitation: 0, specific humidity: 0.0065–0.0067.

The results of real-time prediction in Multan generated in July 2022 are shown in Figure 17d. The disease that occurred in Multan was only malaria. Pink color heatmaps represent malaria. These results of malaria in the Multan district can be verified from Figure 11b. The total no. of mapped locations was 88. At that time, we saved the climate data of the current time, which was as follows: the minimum and maximum temperature was 305.09–312.15 K (31.94–39 °C), precipitation was 0, and minimum to maximum specific humidity was 0.0116–0.0198.

Malaria in Bahawalpur is represented using heatmaps with pink color. The total number of union councils in Bahawalpur was 148. The values of climate features at that time were temperature: 308.91–311.76 K (35.76–38.61 °C), precipitation: 0, humidity: 0.0169–0.0188. Figure 17e shows the results of the Bahawalpur district visualized on WebGIS using machine learning. This result was generated in July 2022. We can verify these results of Malaria from Figure 11b.

## 5. Conclusions

The identification of dengue, malaria, and leishmaniasis breeding sites using spatiotemporal data on a real-time platform has been completed in this research. To predict in real time, there was a need first to train a model on historical data. The dataset consisted of dengue, malaria, leishmaniasis cases, climate, socioeconomic data, and population census. Dengue and leishmaniasis cases were fewer in number due to the class imbalance problem. SMOTE analysis was conducted. These two classes were oversampled according to data sample sizes. Four data samples of different sizes were created to compare the models’ efficiency on that dataset. The data samples were processed with manual feature selection on only climate features to identify which climate factor affected the VBDs the most. The data sample with all features was also used. The results showed that temperature, precipitation, and specific humidity significantly affect the spread of dengue, malaria, and leishmaniasis. Five models for data training—LGBM, RF, DT, SVM, and MLP—were used. The data samples were created to analyze the performance of a model on SMOTE data of different data sizes. To compare which is the best to be used in WebGIS, a comparison of all the results generated from all five models for these data samples has been made. RF is the best-fitted model with 93.97% accuracy achieved on the data sample of 6000, including all features. This accuracy was achieved using a data sample with all climate features, including socioeconomic, population, and patient case data. The data sample 9000 lost accuracy because of much augmentation while using SMOTE, which caused overfitting of the model.

The results showed that LGBM is an efficient model for small datasets, and RF is the best model for large datasets. The best-fitted model was trained and used in WebGIS for real-time predictions of dengue, malaria, and leishmaniasis risk locations in Punjab, Pakistan. In real time, the temperature, precipitation, and relative humidity data were converted into the format according to the training data; then, the website predicted the vulnerable locations in the form of heatmaps on Google Maps.

## 6. Future Work

This research can be extended to other provinces of Pakistan. The dataset from 2014 to 2018 was used, which needed to be updated for new trends of VBDs in Punjab. The climate is changing due to global warming and socioeconomic factors, and population census data should also be updated for 2022. We can extend this work after the addition of the dataset from 2019 to 2023 to the previous dataset. Moreover, we did not use elevation and environmental variables such as NDVI, and these factors also impact on VBDs. This research has used real-time climate data and can use real-time model learning. Furthermore, we can extend this work to alert systems for vaccination, etc., which are interconnected with public health organizations.

## Figures and Tables

**Table 1:** List of Variables of VBDs Dataset.

Sr. No.	Variables	Data Type
1	Disease	Patients Data
2	District	
3	RHC/Name	
4	Latitude	
5	Longitude	
6	Date/Time	
7	Precipitation	Climate Data
8	Temperature	
9	Specific humidity	
10	Access to improved drinking water (% of population)	Socioeconomic Data
11	Access to piped water (% of population)	
12	Access to motorized pump (% of population)	
13	Access to hand pump (% of population)	
14	Access to improved drinking water, excl. piped water (% of population)	
15	Access to improved toilet facilities (% of population)	
16	Access to flush toilet connected to sewer (% of population)	
17	Access to flush toilet connected to septic tank (% of population)	
18	Open defecation (% of population)	
19	Diarrhea incidence (% of children under 5 who had diarrhea during a 2-week recall period)	
20	Using soap for handwashing (% of population)	
21	Using adequately iodized salt (% of population)	
22	Treating water before drinking (% of population)	
23	Population (Population Census 2017—Provisional Results)	Population Data

**Table 2:** Classification Report of Machine Learning Models on All Created Data Sets.

Data Samples	Feature Selection	Machine Learning Models	Diseases	Precision	Recall	F1-Score	Accuracy
Data Sample 2700	Temperature	LGBM	Dengue	92%	95%	93%	90.80%
			Malaria	91%	84%	87%	
			Leishmaniasis	90%	94%	92%	
		RF	Dengue	91%	96%	93%	90.43%
			Malaria	90%	83%	87%	
			Leishmaniasis	90%	92%	91%	
		DT	Dengue	91%	95%	93%	90.56%
			Malaria	91%	84%	87%	
			Leishmaniasis	90%	93%	91%	
		SVM	Dengue	89%	74%	81%	80.37%
			Malaria	71%	80%	75%	
			Leishmaniasis	84%	87%	85%	
		MLP	Dengue	87%	84%	85%	84.81%
			Malaria	81%	79%	80%	
			Leishmaniasis	86%	92%	89%	
	Precipitation	LGBM	Dengue	91%	77%	83%	85.12%
			Malaria	77%	87%	82%	
			Leishmaniasis	89%	91%	90%	
		RF	Dengue	91%	75%	82%	84.01%
			Malaria	75%	87%	81%	
			Leishmaniasis	89%	89%	89%	
		DT	Dengue	90%	75%	82%	83.46%
			Malaria	75%	86%	80%	
			Leishmaniasis	88%	89%	89%	
		SVM	Dengue	89%	73%	80%	79.14%
			Malaria	70%	78%	74%	
			Leishmaniasis	82%	86%	84%	
		MLP	Dengue	86%	78%	82%	81.60%
			Malaria	74%	78%	76%	
			Leishmaniasis	85%	89%	87%	
	Specific Humidity	LGBM	Dengue	91%	94%	92%	90.25%
			Malaria	92%	83%	87%	
			Leishmaniasis	88%	94%	91%	
		RF	Dengue	90%	96%	93%	90.12%
			Malaria	92%	82%	87%	
			Leishmaniasis	88%	93%	91%	
		DT	Dengue	90%	97%	93%	89.44%
			Malaria	93%	78%	85%	
			Leishmaniasis	86%	93%	90%	
		SVM	Dengue	90%	77%	83%	81.30%
			Malaria	73%	80%	76%	
			Leishmaniasis	83%	87%	85%	
		MLP	Dengue	89%	82%	86%	85.37%
			Malaria	79%	83%	81%	
			Leishmaniasis	88%	91%	90%	
	All Features	LGBM	Dengue	91%	97%	94%	91.30%
			Malaria	94%	83%	88%	
			Leishmaniasis	90%	93%	91%	
		RF	Dengue	92%	95%	94%	90.86%
			Malaria	92%	84%	88%	
			Leishmaniasis	89%	94%	91%	
		DT	Dengue	91%	97%	94%	90.74%
			Malaria	92%	83%	87%	
			Leishmaniasis	89%	93%	91%	
		SVM	Dengue	90%	77%	83%	81.17%
			Malaria	72%	81%	76%	
			Leishmaniasis	84%	86%	85%	
		MLP	Dengue	88%	95%	91%	88.58%
			Malaria	90%	80%	85%	
			Leishmaniasis	88%	91%	90%	
Data Sample 3000	Temperature	LGBM	Dengue	90%	95%	93%	90.56%
			Malaria	91%	83%	87%	
			Leishmaniasis	91%	93%	92%	
		RF	Dengue	92%	97%	94%	91.17%
			Malaria	91%	84%	87%	
			Leishmaniasis	91%	92%	92%	
		DT	Dengue	91%	96%	93%	91.06%
			Malaria	91%	84%	87%	
			Leishmaniasis	91%	93%	92%	
		SVM	Dengue	89%	77%	83%	80.28%
			Malaria	72%	77%	74%	
			Leishmaniasis	82%	86%	84%	
		MLP	Dengue	87%	84%	86%	84.67%
			Malaria	79%	80%	80%	
			Leishmaniasis	87%	89%	88%	
	Precipitation	LGBM	Dengue	90%	79%	84%	84.78%
			Malaria	76%	87%	81%	
			Leishmaniasis	89%	89%	89%	
		RF	Dengue	90%	77%	83%	84.06%
			Malaria	76%	87%	81%	
			Leishmaniasis	89%	89%	89%	
		DT	Dengue	90%	77%	83%	84.22%
			Malaria	76%	86%	81%	
			Leishmaniasis	88%	89%	89%	
		SVM	Dengue	87%	78%	82%	79.61%
			Malaria	71%	74%	73%	
			Leishmaniasis	81%	86%	84%	
		MLP	Dengue	89%	77%	83%	81.94%
			Malaria	73%	83%	77%	
			Leishmaniasis	86%	86%	86%	
	Specific Humidity	LGBM	Dengue	88%	96%	92%	89.72%
			Malaria	92%	81%	86%	
			Leishmaniasis	90%	91%	90%	
		RF	Dengue	89%	95%	92%	89.56%
			Malaria	91%	82%	86%	
			Leishmaniasis	90%	91%	90%	
		DT	Dengue	89%	96%	92%	89.61%
			Malaria	91%	81%	86%	
			Leishmaniasis	89%	92%	90%	
		SVM	Dengue	89%	74%	80%	78.28%
			Malaria	69%	74%	71%	
			Leishmaniasis	80%	87%	83%	
		MLP	Dengue	87%	89%	88%	86.17%
			Malaria	83%	80%	81%	
			Leishmaniasis	89%	89%	89%	
	All Features	LGBM	Dengue	92%	97%	95%	91.44%
			Malaria	92%	84%	88%	
			Leishmaniasis	90%	93%	91%	
		RF	Dengue	92%	97%	94%	91.61%
			Malaria	91%	86%	89%	
			Leishmaniasis	91%	92%	92%	
		DT	Dengue	91%	97%	94%	91.33%
			Malaria	93%	84%	88%	
			Leishmaniasis	90%	93%	92%	
		SVM	Dengue	90%	78%	83%	80.78%
			Malaria	72%	78%	75%	
			Leishmaniasis	82%	86%	84%	
		MLP	Dengue	86%	95%	90%	87.50%
			Malaria	88%	79%	84%	
			Leishmaniasis	89%	88%	88%	
Data Sample 6000	Temperature	LGBM	Dengue	94%	96%	95%	92.47%
			Malaria	94%	88%	91%	
			Leishmaniasis	90%	93%	91%	
		RF	Dengue	95%	96%	95%	93%
			Malaria	95%	89%	92%	
			Leishmaniasis	90%	94% 92%		
		DT	Dengue	95%	96%	95%	92.97%
			Malaria	95%	89%	92%	
			Leishmaniasis	89%	94%	92%	
		SVM	Dengue	83%	91%	87%	85.14%
			Malaria	88%	79%	83%	
			Leishmaniasis	85%	85%	85%	
		MLP	Dengue	89% 95%	92%	89.58%	
			Malaria	92%	83%	87%	
			Leishmaniasis	88%	90%	89%	
	Precipitation	LGBM	Dengue	87% 88%	87%	87.06%	
			Malaria	92%	87%	89%	
			Leishmaniasis	83%	86%	85%	
		RF	Dengue	83%	91%	87%	86.25%
			Malaria	92%	84%	88%	
			Leishmaniasis	85%	83%	84%	
		DT	Dengue	81%	92% 86%	85.50%	
			Malaria	93%	80%	86%	
			Leishmaniasis	84%	84%	84%	
		SVM	Dengue	58%	94%	72%	69.92%
			Malaria	90%	36%	51%	
			Leishmaniasis	83%	79%	81%	
		MLP	Dengue	81%	90%	86%	84.53%
			Malaria	86%	84%	85%	
			Leishmaniasis	87%	79%	83%	
	Specific Humidity	LGBM	Dengue	94%	96%	95%	92.47%
			Malaria	94%	88%	91%	
			Leishmaniasis	89%	93%	91%	
		RF	Dengue	91%	95%	93%	90.58%
			Malaria	93%	86%	90%	
			Leishmaniasis	88%	91%	89%	
		DT	Dengue	89%	95%	92%	89.69%
			Malaria	95%	83%	89%	
			Leishmaniasis	86%	91%	88%	
		SVM	Dengue	83%	89%	86%	79.25%
			Malaria	76%	80%	78%	
			Leishmaniasis	79%	68%	73%	
		MLP	Dengue	92%	90%	91%	89.72%
			Malaria	90%	86%	88%	
			Leishmaniasis	87%	92%	90%	
	All Features	LGBM	Dengue	95%	98%	96%	93.39%
			Malaria	96%	89%	92%	
			Leishmaniasis	90%	93%	92%	
		RF	Dengue	96%	97%	97%	93.97%
			Malaria	96%	90%	93%	
			Leishmaniasis	91%	94%	92%	
		DT	Dengue	95%	97%	96%	93.47%
			Malaria	96%	89%	92%	
			Leishmaniasis	89%	94%	92%	
		SVM	Dengue	82%	91%	86%	83.72%
			Malaria	86%	79%	82%	
			Leishmaniasis	84%	81%	82%	
		MLP	Dengue	91%	94%	93%	90.36%
			Malaria	93%	84%	88%	
			Leishmaniasis	87%	93%	90%	
Data Sample 9000	Temperature	LGBM	Dengue	90%	97%	93%	91.50%
			Malaria	94%	84%	89%	
			Leishmaniasis	91%	94%	92%	
		RF	Dengue	91%	97%	94%	91.76%
			Malaria	94%	85%	89%	
			Leishmaniasis	91%	94%	92%	
		DT	Dengue	90%	97%	93%	91.61%
			Malaria	94%	84%	89%	
			Leishmaniasis	91%	94%	92%	
		SVM	Dengue	69%	82%	75%	74.94%
			Malaria	72%	54%	62%	
			Leishmaniasis	84%	89%	86%	
		MLP	Dengue	84%	96%	89%	87.87%
			Malaria	92%	76%	83%	
			Leishmaniasis	89%	92%	91%	
	Precipitation	LGBM	Dengue	91%	79%	85%	86.09%
			Malaria	78%	88%	83%	
			Leishmaniasis	90%	91%	91%	
		RF	Dengue	90%	79%	84%	85.42%
			Malaria	78%	86%	82%	
			Leishmaniasis	89%	91%	90%	
		DT	Dengue	89%	79%	84%	84.19%
			Malaria	77%	82%	80%	
			Leishmaniasis	87%	91%	89%	
		SVM	Dengue	63%	90%	74%	71.17%
			Malaria	74%	35%	47%	
			Leishmaniasis	81%	88%	84%	
		MLP	Dengue	90%	77%	83%	83.67%
			Malaria	75%	84%	80%	
			Leishmaniasis	87%	90%	88%	
	Specific Humidity	LGBM	Dengue	90%	97%	93%	91.28%
			Malaria	94%	83%	88%	
			Leishmaniasis	91%	94%	92%	
		RF	Dengue	89%	94%	92%	89.92%
			Malaria	91%	82%	86%	
			Leishmaniasis	90%	93%	91%	
		DT	Dengue	89%	97%	93%	89.81%
			Malaria	93%	79%	86%	
			Leishmaniasis	88%	93%	91%	
		SVM	Dengue	64%	90%	75%	71.06%
			Malaria	74%	33%	45%	
			Leishmaniasis	79%	90%	84%	
		MLP	Dengue	81%	95%	88%	86.87%
			Malaria	90%	74%	81%	
			Leishmaniasis	90%	91%	91%	
	All Features	LGBM	Dengue	91%	98%	95%	92.42%
			Malaria	95%	85%	90%	
			Leishmaniasis	91%	94%	93%	
		RF	Dengue	92%	98%	95%	92.96%
			Malaria	96%	86%	91%	
			Leishmaniasis	92%	94%	93%	
		DT	Dengue	92%	98%	95%	92.63%
			Malaria	95%	85%	90%	
			Leishmaniasis	91%	94%	93%	
		SVM	Dengue	76%	88%	82%	80.31%
			Malaria	80%	62%	70%	
			Leishmaniasis	84%	91%	88%	
		MLP	Dengue	87%	97%	92%	89.87%
			Malaria	93%	80%	86%	
			Leishmaniasis	90%	92%	91%	

## Data Availability

Not applicable.
