# Correction: Javaid et al. WebGIS-Based Real-Time Surveillance and Response System for Vector-Borne Infectious Diseases. *Int. J. Environ. Res. Public Health* 2023, *20*, 3740

**DOI:** 10.3390/ijerph22040499

**Published:** 2025-03-26

**Authors:** Momna Javaid, Muhammad Shahzad Sarfraz, Muhammad Umar Aftab, Qamar uz Zaman, Hafiz Tayyab Rauf, Khalid A. Alnowibet

**Affiliations:** 1 Department of Computer Science, National University of Computer and Emerging Sciences, Islamabad, Chiniot-Faisalabad Campus, Chiniot 35400, Pakistan; 2 Independent Researcher, Bradford BD8 0HS, UK; 3 Statistics and Operations Research Department, College of Science, King Saud University, Riyadh 11451, Saudi Arabia

In the published publication [1], there was an error regarding the affiliation of Hafiz Tayyab Rauf. The updated affiliation should be: Independent Researcher, Bradford BD8 0HS, UK. The authors state that the scientific conclusions are unaffected. This correction was approved by the Academic Editor. The original publication has also been updated.
